# Calcium ions in the aquatic environment drive planarians to food

**DOI:** 10.1186/s40851-019-0147-x

**Published:** 2019-11-06

**Authors:** Masato Mori, Maria Narahashi, Tetsutaro Hayashi, Miyuki Ishida, Nobuyoshi Kumagai, Yuki Sato, Reza Bagherzadeh, Kiyokazu Agata, Takeshi Inoue

**Affiliations:** 1 Department of Life Science, Faculty of Science, Gakushuin University, 1-5-1 Mejiro, Toshima-ku, Tokyo, Japan; 2 Laboratory for Bioinformatics Research, RIKEN Center for Biosystems Dynamics Research, 2-2-3 Minatojima-Minamimachi, Chuo-ku, Kobe, Japan; 3 Department of Biophysics, Graduate School of Science, Kyoto University, Kitashirakawa-Oiwake, Sakyo-ku, Kyoto, Japan; 4 Department of Stem Cells and Developmental Biology, Cell Science Research Center, Royan Institute for Stem Cell Biology and Technology, Banihashem, Tehran, Iran; 5 Department of Developmental Biology, University of Science and Culture, Banihashem, Tehran, Iran; 6 National Institute for Basic Biology, National Institutes of Natural Science, 38 Nishigonaka, Myodaiji, Okazaki, Japan

**Keywords:** Planarian, Environmental changes, Quantitative behavioristics, Feeding behavior, Environment-responsive behavior, Responsive sensitivity

## Abstract

**Background:**

Even subtle changes in environmental factors can exert behavioral effects on creatures, which may alter interspecific interactions and eventually affect the ecosystem. However, how changes in environmental factors impact complex behaviors regulated by neural processes is largely unknown. The freshwater planarian *Dugesia japonica*, a free-living flatworm, displays distinct behavioral traits mediated by sensitive perception of environmental cues. Planarians are thus useful organisms for examining interactions between environmental changes and specific behaviors of animals.

**Results:**

Here we found that feeding behavior was suppressed when the concentration of ions in the breeding water was low, while other behaviors were unaffected, resulting in differences in population size. Notably, the decline in feeding behavior was reversed in an ion-concentration-dependent manner soon after the planarians were moved to ion-containing water, which suggests that ions in environmental water rapidly promote feeding behavior in planarians. Moreover, the concentration of ions in the environmental water affected the feeding behavior by modulating the sensitivity of the response to foods. Finally, we found that calcium ions in the aquatic environment were required for the feeding behavior, and exposure to higher levels of calcium ions enhanced the feeding behavior, showing that there was a good correlation between the concentration of calcium ions and the responsiveness of planarians to foods.

**Conclusions:**

Environmental calcium ions are indispensable for and potentiate the activity level of the feeding behavior of planarians. Our findings suggest that the ions in the aquatic environment profoundly impact the growth and survival of aquatic animals via modulating their neural activities and behaviors.

## Background

Environmental changes affect homeostasis, growth, reproduction, and other essential biological functions, and animals sense environmental conditions and accordingly adopt survival strategies, such as activity, migration, reproductive behavior, and morphological alteration. Physiological end effects in animals, such as reproductive defects or behavioral disorders, caused by environmental factors such as pollutants or toxins have been intensively studied; however, how changes of natural environmental factors influence common animal behaviors, which are regulated by complex physiological and neural processes, remains poorly understood despite their importance for animal ethology, ecology, and evolution.

The planarian *Dugesia japonica*, a free-living flatworm, inhabits clear freshwater, such as rivers and lakes [1], and planarians are susceptible to toxins or pollutants in their aquatic environment. Therefore, planarians have been used as an indicator species in the fields of ecology and toxicology to assess the effect of water pollutants on aquatic animals [2, 3]. Moreover, planarians are well known for their remarkable regenerative ability [4–6]. Due to their unique physiological traits and phylogenetic position, planarians have been extensively used as experimental animals in the laboratory for investigations in a wide variety of biological fields, such as regenerative machinery, dynamics of stem cells, organogenesis, neuroscience, reproductive strategy, and animal evolution. In such investigations, various sustainable culture methods have been used, such as culturing in dechlorinated tap water [7], artificial culture solution [8, 9], or diluted artificial seawater [10, 11]. It was previously found that calcium, potassium, and sodium ions are important for culturing planarian cells and tissues [8, 12, 13], and remarkably, planarians can survive for a long time in a solution containing only calcium chloride [14], indicating that calcium ions are sufficient for planarian survival. In addition, lithium induces the formation of supernumerary eyes, one of the most evident malformations in planarians. The degree of this lithium-induced malformation is modulated by the competitive effects of sodium and potassium ions in the water in which planarians live [15]. These findings suggest that ions in the environmental water may impact planarian physiology; however, how ions in the aquatic environment affect planarian behaviors subject to neural regulation remains obscure.

The central nervous system of planarians is composed of a bi-lobed brain in the anterior region of the animal and two longitudinal ventral nerve cords along the body [16, 17]. Recent molecular studies have demonstrated that, despite their simple body pattern, planarians have a sophisticated brain [18, 19]. Furthermore, studies using behavioral assays quantifying complex behaviors demonstrated that planarians display multiple environment-responsive behaviors mediated by the sensing of environmental signals, such as light, soluble chemicals, and temperature, via distinct sensory organs and integrating them in the brain [20–24]. Among their environment-responsive behaviors, planarians’ feeding is a complex behavior that can be divided into distinct processes. Planarians ingest food via the pharynx, which is protruded out from the middle portion of the body, after localization of the target food using soluble substances released from foods as the clue [25, 26]. Despite the variety of available quantitative behavioral assays [20–24], a method to quantify the food intake as the endpoint of the feeding behavior is still lacking. Recently, the usefulness of the visualization of colored chalks ingested together with food and double-stranded RNA for achieving stable RNAi gene knockdown in planarians was described [27], which suggests that feeding of colored chalks plus food may help to quantify the amount of food ingested. These findings indicate that behavioral assays in planarians offer unique opportunities for investigating animal behaviors associated with environmental changes. Here we investigated long-term and acute effects on planarian feeding behavior in aquatic environments with different ion compositions.

## Methods

### Waters

Highly purified water (pure water) (18 MΩ cm resistivity)(Merck), diluted artificial seawater (ASW) (Instant Ocean Sea Salt; Spectrum Brands), dechlorinated tap water (Bureau of Waterworks, Tokyo Metropolitan Government), and Kanatani’s planarian breeding water (hereafter referred to as “Kanatani water”) (0.77 mM CaCl_2_, 0.07 mM KCl, 0.64 mM NaCl, 0.17 mM NaHCO_3_, pH 7.6) [8] were used. The ion composition of modified Kanatani water used in this study is shown in Additional file 1: Table S1. ASW was prepared by dilution with pure water (Merck). Dechlorination of tap water was performed by autoclaving or treatment with sodium thiosulfate.

### Animals

Clonal strains of planarians (SSP-9 T strain of *D. japonica* [28] or BCN-10 strain of *Schmidtea mediterranea* [29]) were used in all experiments.

### Breeding test

Ten planarians were cultured in a 90-mm-diameter plastic Petri dish containing 40 ml of a breeding water (Kanatani water, tap water, 0.05% ASW, 0.005% ASW, or 0% ASW/pure water) for 6 weeks. They were fed chicken liver twice a week and were cultured under conditions in which there was no competition for space or food, as referred to in previous reports [30, 31]. Breeding waters were changed every 2 days. Planarians that were 7-mm-long along the anterior-posterior axis and that had been derived from one culture tank and had been starved for 1 week were used as starting animals for behavioral experiments.

### Assays of planarian behaviors

All behavioral experiments were performed in a dark room with only a red light, the wavelength of which does not induce a behavioral response by planarians [32–34]. Planarians were kept in the dark for at least 60 min in breeding water before the experiment. For the food-intake assay, planarians were put into a 90-mm-diameter plastic Petri dish filled with test water, and allowed to feed on colored food pellets containing the pink-colored chalk powder [27] for 30 min. The colored food pellet was prepared as a mixture of 10 μL of chalk powder solution, 25 μL (62.5%) of chicken liver homogenate, and 5 μL of 2% agarose. To quantify the intake of the food, fed planarians were put on ice and photographed under a stereoscopic microscope (Leica M205 FA) with bright field illumination to visualize the planarian shape and a Texas Red filter set. Fluorescence was quantified using Fiji/ImageJ and fluorescence intensity was expressed as the food intake after binarization with a certain threshold. Feeding index was calculated using Eq. 1:

1 $$ Feeding\ index={A}_f/{A}_w $$

$$ {A}_f: The\ area\ of\ fluorescence $$

$$ {A}_w: The\ projected\ area\ of\ the\ whole\ body $$

For the food-localization behavior assay, planarians were put in a 90-mm-diameter plastic Petri dish filled with test water, and a target food was put at the center of the dish. As a target food, a 5-mm cube of cut chicken liver or a food pellet was used. Planarian behavior was recorded for 5 or 10 min using a camera (Sony ILCE-7) fixed above the assay field. Trajectories were tracked every 200 msec, and the data were analyzed using a computer, Fiji/ImageJ, and R software v.3.5.1 (R Foundation for Statistical Computing, Vienna, Austria). The speed of an individual was calculated as the median value of the score measured every 200 msec during an assay.

For the photo-orientation behavior assay, planarians were put into a 60 × 30 × 10 mm container filled with test water [35]. The container was exposed to 500 lx of white light emitted from a horizontally positioned light source and passed through a UV-cut filter. Planarian behavior was recorded for 90 s. The time animals spent in the target quadrant located opposite to the light source during the test was analyzed. The data were analyzed using the same method as used for the food-localization behavior assay.

To remove cilia of planarians, individuals were soaked in 1% ethanol in Kanatani water for 10 min at room temperature as previously described [36, 37], and subsequently planarians were used for behavioral assays in test waters, which contained no ethanol.

### Live imaging of motile cilia

For live imaging of cilia in the peripheral epithelium, planarians were placed in a particular test water on a glass microscope slide attached to a perforated sheet of Parafilm and capped with a slide cover, as described previously [38, 39]. Movement of cilia was imaged by phase contrast microscopy on a Nikon Eclipse Ts2 microscope. Video segments were acquired at 240 frames/seconds and kymographs were produced from line scans perpendicular to the row of cilia using ImageJ/Fiji.

### The ion composition of waters

For calculation of the concentration of calcium ions, potassium ions, and sodium ions in the Kanatani water (Additional file 1: Table S1), the tap water used in this study, and rivers in Japan, we used published data [8, 40, 41]. For selectively chelating calcium ions, ethylene glycol tetra-acetic acid (EGTA) was added at a final concentration of 10 mM to Kanatani water or tap water.

### Statistical analysis

Data were analyzed by one-way analysis of variance (ANOVA), the Kruskal-Wallis test, or Dunnett test for overall group comparisons. For calculation of the difference from the normal distribution, the Kolmogorov-Smirnov test was used. The F-test was used to analyze normally distributed values. The statistical significance of differences between test results was determined by the Wilcoxon rank sum test or Welch two-sample t-test. Optionally, Holm correction was used to adjust significance levels for multiple comparisons. All tests were conducted using R software. Significance was defined as *p* < 0.05.

## Results

### Breeding water affects the feeding behavior

**Fig. 1:**
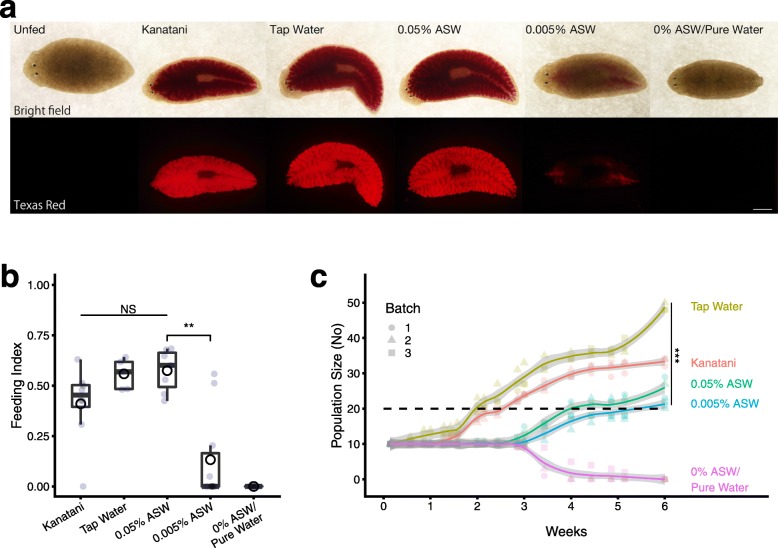
Breeding water affects food intake and population size. **a**. Typical living animals that had been cultured in five kinds of breeding water and had then ingested liver homogenate tagged with a pink powder. Ingested food was visualized using a Texas Red fluorescence filter set. No fluorescence signals were detected in the unfed planarians. The strong fluorescence signals were detected in the planarians grown in Kanatani water, tap water, or 0.05% ASW, while weak signals were detected in planarians grown in 0.005% ASW or 0% ASW/pure water. Anterior is to the left. Bar, 1 mm. All animals are shown from the dorsal side. **b**. The feeding index determined by the ratio between the fluorescence area and the whole body area is shown as a box-and-whisker plot with mean (open circles). The feeding index of planarians grown in Kanatani water, tap water, or 0.05% ASW showed high score and there were no significant differences (NS by Kruskal-Wallis test) among them. By contrast, a low concentration (0.005% ASW) or absence (0% ASW/pure water) of ions in the breeding water reduced the score of the feeding index. **, *p* < 0.01 (Wilcoxon test). **c**. Population growth curve of planarians cultured in breeding waters for 6 weeks. The curves are shown as a trend line of three independent batches with 95% confidence interval (gray). The horizontal dashed line indicates the doubling of the number of individuals present at the start point. The number of individuals at 6 weeks was different, depending on the breeding water. ***, *p* < 0.005 (ANOVA)

In order to examine the food intake of planarians in various different breeding waters, we performed a food-intake assay that visualizes ingested food as a fluorescent signal by using the powder of pink-colored chalk as food coloring [27]. A colored food pellet was supplied to planarians in each of three different kinds of commonly used breeding waters: Kanatani’s planarian breeding water (Kanatani water) [8], dechlorinated tap water (tap water) [7], and 0.005% artificial seawater (ASW) [11]. Additionally, two other different concentrations of ASW, namely 0.05% ASW and 0% ASW/pure water, were examined. The results showed that although planarians cultured in Kanatani water, tap water, and 0.05% ASW ingested substantial amounts of foods, the food intake of planarians grown in 0.005% ASW and 0% ASW/pure water was much lower (Fig. 1a). To quantify the food intake, we defined the feeding index as the ratio between the fluorescent area of the ingested food and the whole body area. Comparison of the feeding indexes indicated that a low concentration (0.005% ASW) or the absence (0% ASW/pure water) of ions significantly reduced the food intake, although there were no significant differences among planarians in the other breeding waters examined (Fig. 1b). These results indicated that the food intake of planarians was different depending on the breeding water, and was suppressed when there was a low concentration of ions in the breeding water.

To examine the proliferation of planarians as an outcome of nutrient intake, 10 individuals taken simultaneously from a population clonally cultured in a breeding tank were reared for 6 weeks with regular feedings in the respective breeding waters. The results shown in Fig. 1c indicate that the number of individuals increased monotonically to three (or more) times the starting number during 6 weeks of cultivation in tap water, Kanatani water, or 0.05% ASW in three independent batches; however, the number of individuals cultured in 0.005% ASW only approximately doubled during the 6-week period, and all of the planarians cultured in 0% ASW/pure water remained alive for 3 weeks but died without proliferation by 6 weeks (Fig. 1c). Planarians can generally survive for several months without feeding, and therefore the death of the planarians in 0% ASW/pure water might have been caused by the homeostatic state resulting from the extremely low concentration of salts in the breeding water, rather than by the loss of feeding. The doubling time (indicated by the horizontal dashed line in Fig. 1c) of individuals bred in Kanatani water or tap water was less than 2 weeks during the test. The doubling time of individuals bred in 0.005% ASW was 6 weeks, and increasing the concentration of ASW to 0.05% shortened the doubling time to 4 weeks. Statistical analysis at 6 weeks indicated that the population size was significantly different depending on the breeding water, and that the ion concentration in the breeding water affected the proliferation of planarians. These observations from breeding experiments suggest that reduction of the concentration of ions in the breeding water correlates with reduction of the food intake, resulting in a reduction of the population size of planarians.

**Fig. 2:**
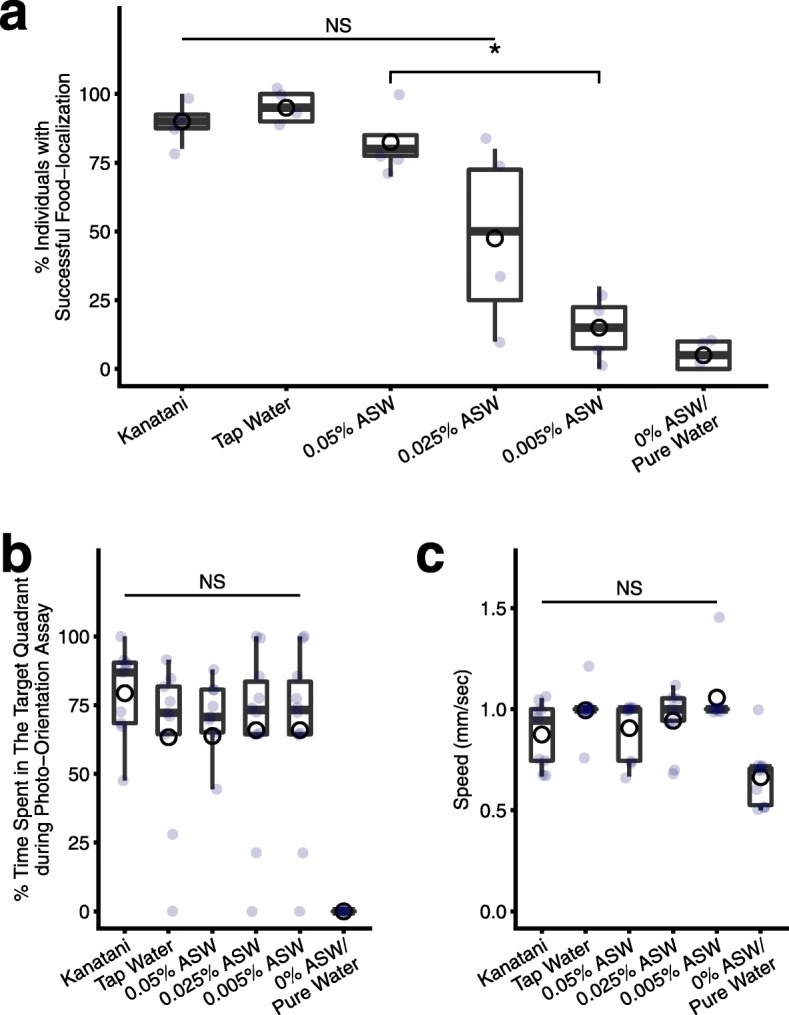
Breeding water affects the food-localization behavior. **a**. The percent of individuals that reached the food (piece of liver) within 10 min is shown as a box-and-whisker plot with mean (open circles). *n* = 10; 4 independent experiments. Although there were no significant differences (NS by Kruskal-Wallis test) among planarians grown in Kanatani water, tap water, 0.05% ASW, and 0.025% ASW (Kruskal-Wallis test), the food-localization of planarians cultured in breeding water containing a lower concentration (0.005% ASW) or absence (0% ASW/pure water) of ions was suppressed. *, *p* < 0.05 (Wilcoxon test). **b**. The time spent in the target quadrant during the photo-orientation assay. Only the planarians that were grown in the 0% ASW/pure water showed impairment of the photo-orientation behavior, while the others were unaffected. There were no significant differences (NS) among planarians grown in Kanatani water, tap water, 0.05% ASW, 0.025% ASW, and 0.005% ASW (Kruskal-Wallis test). **c**. The motor activity indicated by the average speed. Although the speed of planarians grown in the pure water was slightly decreased, the others were unaffected. There were no significant differences (NS) among the speeds of planarians grown in Kanatani water, tap water, 0.05% ASW, 0.025% ASW, and 0.005% ASW (Kruskal-Wallis test)

To examine the involvement of the feeding behavior in the difference of food intake in different breeding waters, we performed a food-localization behavior assay. The results indicated that the fraction of individuals that reached food within 10 min was different depending on the breeding water (Fig. 2a). Planarians cultured in Kanatani water, tap water, or 0.05% ASW all showed a similar level of activity of food-localization behavior, whereas planarians cultured in a low concentration (0.005% ASW) or the absence (0% ASW/pure water) of ions showed a decreased score of food-localization behavior, and planarians cultured in an intermediate concentration of ions (0.025% ASW) showed highly variable scores of food-localization behavior (Fig. 2a), indicating that feeding behavior may be dependent on breeding water properties, and that a low concentration or absence of ions in the breeding water may inhibit the feeding behavior.

To examine whether the low concentration or absence of ions in the breeding water specifically suppresses the feeding behavior, or whether it suppresses the overall behavioral capacities of planarians, we performed a photo-orientation assay using planarians after 2 weeks of cultivation in the respective breeding waters. Figure 2b shows the time spent in the target quadrant (the area opposite to the light source) during the photo-orientation assay using a rectangular assay field [35]. Although planarians cultured in 0% ASW/pure water showed a reduction of light-avoidance behavior, we could not find any difference among the photo-orientation behavior of planarians cultured with a high concentration of ions in the breeding water (0.05% ASW), with an intermediate concentration of ions in the breeding water (0.025% ASW), and with a low concentration of ions in the breeding water (0.005% ASW) (Fig. 2b), and these values were comparable to those obtained in Kanatani water and tap water. Moreover, when we compared the speed of planarians’ movement in mm/sec, we could not find any difference among planarians cultured in Kanatani water, tap water, 0.05% ASW, 0.025% ASW, and 0.005% ASW, although impairment of the motor activity was observed in planarians cultured in 0% ASW/pure water (Fig. 2c). These results indicate that long-term breeding in the absence of any ions (0% ASW/pure water) reduces the motor activity of planarians, which might cause reduced scores in the feeding behavior and photo-orientation behavior assays; but the impairment of the feeding behavior found in planarians cultured in breeding water with a low concentration of ions (0.005% ASW) appeared not to be caused by reduced motor activity. These data indicate that the impairment of the overall behavioral activity of planarians in the absence of any ions (0% ASW/pure water) in the breeding water may have been caused by the resultant homeostatic state, whereas no clear impairment of overall behavioral activity was detected in planarians cultured in a low concentration of ions (0.005% ASW). Therefore, our data suggest that the feeding behavior was specifically inhibited by a low concentration of ions in the breeding water.

### The environmental water immediately affects the feeding behavior

**Fig. 3:**
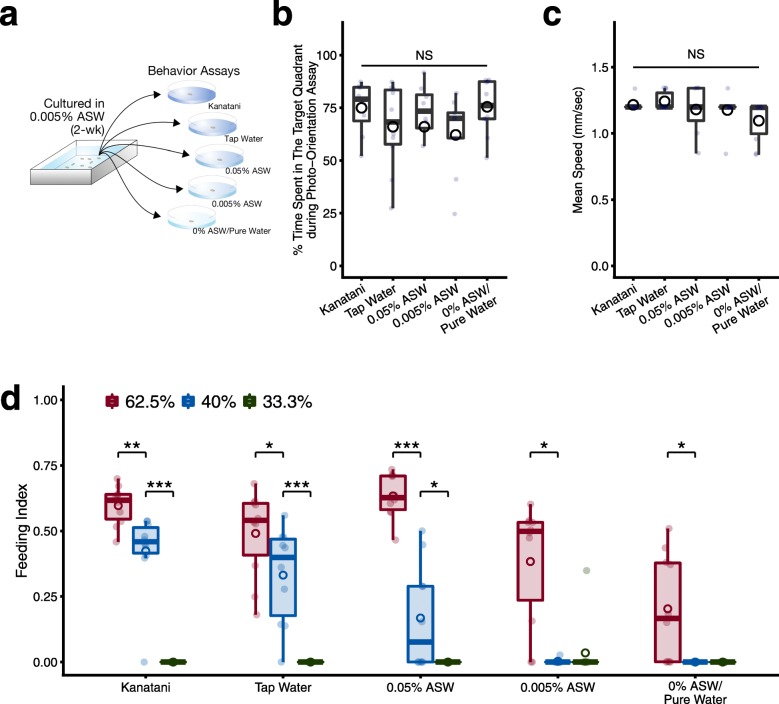
Environmental water immediately affects and can restore feeding behavior specifically. **a**. A schematic illustration of the experimental design for behavioral assays in various environmental waters. Planarians grown in 0.005% ASW for at least 2 weeks were transferred to assay fields containing each type of environmental water and briefly subjected to behavioral assays. **b**. The percent time spent in the target quadrant during 90 s in the photo-orientation assay is shown as a box-and-whisker plot with mean (circles). **c**. Speed of movement (mm/sec) during photo-orientation assay. There were no significant differences (NS) in either the photo-orientation behavior or the motor activity among planarians bred in the different environmental waters (Kruskal-Wallis test). **d**. The concentration of ions in the environmental water determines planarians’ sensitivity to the presence of food. The food intake was analyzed using colored food pellets containing different proportions of liver homogenate (33.3, 40, and 62.5%). Planarians cultured long-term with a low concentration of ions (0.005% ASW) showed restoration of the feeding behavior immediately after transfer into Kanatani water, tap water, or 0.05% ASW. The feeding index increased in a food-proportion-dependent manner. *, *p* < 0.05; **, *p* < 0.01; ***, *p* < 0.005 (Wilcoxon test)

Next, in order to investigate whether some defect of the homeostatic state during longterm culture in breeding water with a low concentration or the absence of ions affects the feeding behavior, or whether the concentration of ions in the breeding water has an immediate effect on the feeding behavior, we transferred planarians that had been bred together in a culture container with a low concentration of ions (0.005% ASW) into assay chambers containing different types of environmental waters and promptly performed behavioral assays (Fig. 3a). First we performed the photo-orientation assay, but we could not find any differences in the photo-orientation behavior or motor activity among planarians in any of the tested environmental waters, including 0% ASW/pure water (Fig. 3b, c). Therefore, the absence of ions in the planarians’ culture water (0% ASW/pure water) might cause a chronic impairment of normal movement (Fig. 2); however, replacement of the culture water or acute changes of osmotic pressure by any of the environmental waters during the photo-orientation assay did not affect the photo-orientation behavior or movements, at least during the assay times used here.

By contrast, the food intake differed depending on the environmental water used in the feeding behavior assay (Fig. 3d). Even planarians cultured long-term with a low concentration of ions (0.005% ASW) showed a higher score of food intake immediately after transfer into Kanatani water, tap water, or 0.05% ASW (Fig. 3d), and their scores were comparable to those of planarians cultured long-term in tap water, Kanatani water or breeding water with a higher concentration of ions (0.05% ASW) (Fig. 1b).

Meanwhile, we found that the food intake of planarians was increased in a food-dose-dependent manner (Fig. 3d). When we used food containing 62.5% liver homogenate, planarians tested in all environmental waters ingested the food. However, when we reduced the concentration of the liver homogenate in the target food to 40%, the feeding indexes of planarians in tap water, Kanatani water, and 0.05% ASW were drastically reduced, and planarians assayed in 0.005% ASW or 0% ASW/pure water failed to consume the food. Furthermore, the planarians tested in any environmental water did not ingest the food containing 33.4% liver homogenate. This result suggests that ions in the environmental water modulate the sensitivity of the responsiveness to foods that produces feeding behavior of planarians.

**Fig. 4:**
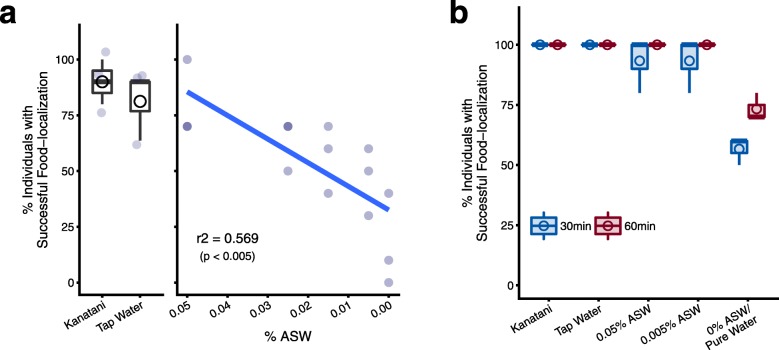
The environmental water affects the sensitivity of the response to foods. **a**. The percent of individuals that reached the food within 10 min. Although the food-localization behavior of planarians tested in Kanatani water, tap water, or 0.05% ASW showed a high score, the scores of the food-localization were gradually decreased according to the reduction of the concentration of ions in the environmental water. Regression analysis showed a good correlation between the food-localization behavior and the concentration of the ions. *n* = 10; 3 independent experiments. **b**. The percent of individuals that reached the food within 30 or 60 min. Prolongation of the assay time resulted in improvement of the score of the food-localization behavior, indicating that low concentrations of ions in the environmental water may reduce the ability to elicit the food-localization behavior

Similarly, we analyzed the food-localization behavior using planarians that were transferred into assay chambers containing different types of environmental waters. The results showed high scores of the food-localization of planarians in Kanatani water and tap water; however, as we decreased the concentration of ions of the environmental water using five different concentrations (0.05, 0.025, 0.015, 0.005, and 0%) of ASW, the scores of the food-localization were gradually decreased (Fig. 4a). Regression analysis between the concentration of ions and the score of food-localization showed a good correlation (*r*^*2*^ = 0.569), indicating that the movement of planarians toward the food tended to be enhanced in an ion-concentration-dependent manner. These data indicate that replacement of the environmental water immediately affected the feeding behavior of even planarians cultured long-term in breeding water with a low concentration of ions (0.005% ASW), and the activity of the feeding behavior was recovered in an ion-concentration-dependent manner. In addition, when we continued to observe the food-localization behavior for a longer time, the score of food-localization was elevated in planarians assayed in all environmental waters after 30 min, and nearly all individuals reached the food after 60 min (Fig. 4b). This result suggests that culturing planarians with a low concentration of ions in the environmental water did not cause complete loss of feeding behavior ability, but rather reduced the efficiency of the feeding behavior. Taken together, our findings indicate that an appropriate concentration of ions in the environmental water used for the food-localization assay, rather than effects of ions on developmental processes or the homeostatic state of planarians, may be required for the feeding behavior, and ions in the environmental water may modulate the sensitivity of planarians’ responsiveness to food.

### Calcium ions in the environmental water are required for the feeding behavior

**Fig. 5:**
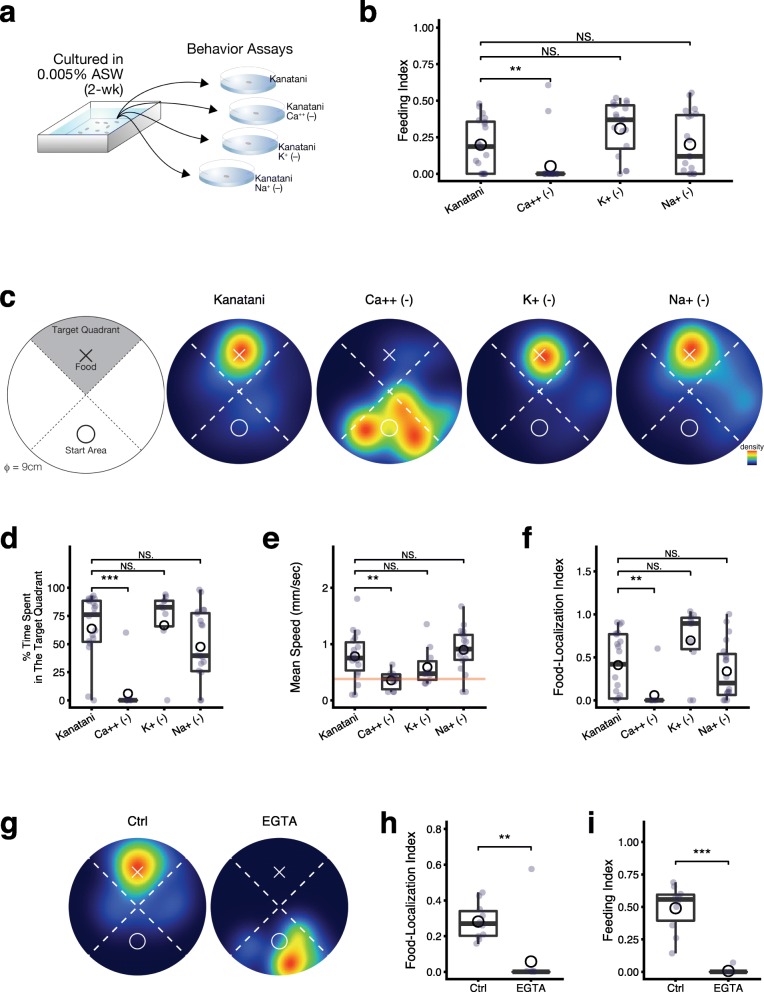
Calcium ions are required for the feeding behavior. **a**. Schematic illustration of the experimental design for the behavioral assays in environmental waters in the absence of a particular ion. Planarians grown in 0.005% ASW for at least 2 weeks were transferred to assay fields containing each type of modified Kanatani water and briefly subjected to behavioral assays. **b**. The feeding index of planarians in Kanatani water in the absence of a particular ion is shown as a box-and-whisker plot with means (circles). The absence of calcium ions in the environmental water reduced food intake, whereas the absence of potassium ions or sodium ions did not affect it. **c**. The food-localization assay in each type of modified Kanatani water. The cross indicates the position of the colored food pellet. The circle indicates the planarian’s start region. Planarian behavior was quantified using the time spent in the target quadrant. The diameter of the circular field was 9 cm. Heat map view of the averaged behavior of 10 individually assayed animals in a food-localization assay field. Warm colors indicate locations where much time was spent, and cool colors those where little time was spent. Planarians in Kanatani water, Kanatani water without potassium ions, or Kanatani water without sodium ions showed a preference for moving to and remaining in the region with the food, whereas planarians in Kanatani water without calcium ions did not show such food-localization behavior. *t* = 300 s. **d**. Time spent in the target quadrant during assay of planarians in Kanatani water without a particular ion is shown as a box-and-whisker plot with mean (circles). **e**. Speed of movement of planarians during the assay. The absence of calcium ions in the environmental water impaired the motor activity. **f**. The food-localization index is the adjusted value of the spent time in the target quadrant calculated by assuming all individuals had the same speed. The adjustment was performed using the median value of the speed (0.38 mm/sec) of the planarians in Kanatani water without calcium ions. **g**. The food-localization assay in Kanatani water containing 10 mM EGTA. Planarians in Kanatani water (Ctrl) showed a preference for moving to and staying in the region with the food, whereas planarians in Kanatani water containing EGTA did not show such food-localization behavior. *t* = 300 s. **h**. The food-localization index is the adjusted value of the spent time in the target quadrant calculated by assuming all individuals had the same speed of movement. The adjustment was performed using the median value of the speed (0.51 mm/sec) of the planarians in Kanatani water containing EGTA. **i**. The feeding index of planarians in Kanatani water containing EGTA is shown as a box-and-whisker plot with means (circles). The chelation of calcium ions by EGTA in the Kanatani water reduced the food intake. *, *p* < 0.05; **, *p* < 0.01; ***, *p* < 0.005; NS, not significant (Wilcoxon test)

In order to gain a better understanding of the relationship between the environmental water and the feeding behavior in planarians, we sought to identify the species of ion(s) required for the feeding behavior in planarians. Kanatani water is a defined artificial breeding water of planarians, and we prepared modified defined waters by subtracting calcium, potassium, or sodium ions from the original Kanatani water. We performed the food-intake assay soon after the transfer of planarians that had been bred in a low concentration of ions (0.005% ASW) into assay chambers containing these modified defined waters (Fig. 5a). The feeding index showed that absence of calcium ions in the environmental water during the assay reduced the food intake, whereas the absence of potassium or sodium ions did not affect the food intake (Fig. 5b). This result indicates that calcium ions in the environmental water are indispensable for ingesting foods.

In addition to using these modified defined waters during the food-intake assay, we devised a tracking assay to measure and quantify in more detail the food-localization behavior, as shown in Fig. 5c. The results of the food-localization assay shown as the averaged movements of 10 independent individuals together with a heat map for these movements indicated that planarians tested in Kanatani water tended to move toward the quadrant where target food was located, indicated by a white cross, which was consistent with the above results of the food-intake assay (Fig. 5c). However, planarians assayed in the Kanatani water lacking calcium ions showed defects in their ability to move toward the food, and instead showed random movements around the start region. In contrast, planarians tested in the Kanatani water without potassium or sodium ions moved toward the food, like planarians in the original Kanatani water. Quantification of the time spent in the target quadrant by these animals clearly indicated that calcium ions, but not potassium or sodium ions, in the environmental water are required for the food-localization behavior (Fig. 5d).

In order to confirm that this requirement for calcium ions did not arise from an effect on the speed of movement, we compared the speed of movement among planarians in the respective waters. The results showed that the absence of calcium ions in the environmental water did in fact slightly impair the speed of movement (Fig. 5e). Therefore, we further evaluated the time spent in the target quadrant as the food-localization index, calculated by assuming that all individuals had the same speed by compensating with the median value of the speed (0.38 mm/sec) of the planarians in the Kanatani water in the absence of calcium ions. The compensated value of food-localization behavior expressed as the food-localization index showed that absence of calcium ions in the environmental water during the assay reduced the activity of food-localization behavior, whereas the absence of potassium or sodium ions did not affect the food-localization behavior (Fig. 5f). These results suggest that absence of calcium ions in the environmental water also reduces motor activity; however, compensating for the reduced motor activity showed that the low score of the time planarians spent in the target quadrant appeared not to be caused by the decrease of the speed due to the absence of calcium ions in the environmental water. Consistent with these results, the ability to move toward the target food indicated by the food-localization index and the food intake indicated by the feeding index were impaired in planarians assayed in Kanatani water or tap water supplemented with EGTA to chelate calcium ions (Fig. 5g-i, Additional file 2: Figure S1).

Planarians exhibit a gliding mode of movement using motile cilia in the peripheral epithelium throughout the body, as well as a crawling mode of movement using muscles, during locomotion. In order to investigate whether the absence of calcium ions in the environmental water affects ciliary beating, we observed ciliary beating in the peripheral epithelium in each environmental water tested here, and could not find any defect of the beating in any of the waters (Additional file 3: Figure S2a). Furthermore, we performed the food-localization assay using planarians whose cilia were removed by treatment with 1% ethanol, and the results showed that the absence of cilia did not affect the activity of moving toward the food, although the speed of moving was slowed due to the loss of gliding ability (Additional file 3: Figure S2b-f). These findings indicated that the absence of calcium ions in the environmental water does not affect the ciliary beating, and motile cilia are not required for the activity of producing the feeding behavior. Taken together, our data suggest that calcium ions in the environmental water were required for the feeding behavior independent on the requirement for calcium for muscle activity in planarians.

### Calcium ions in the environmental water boost the feeding activity

**Fig. 6:**
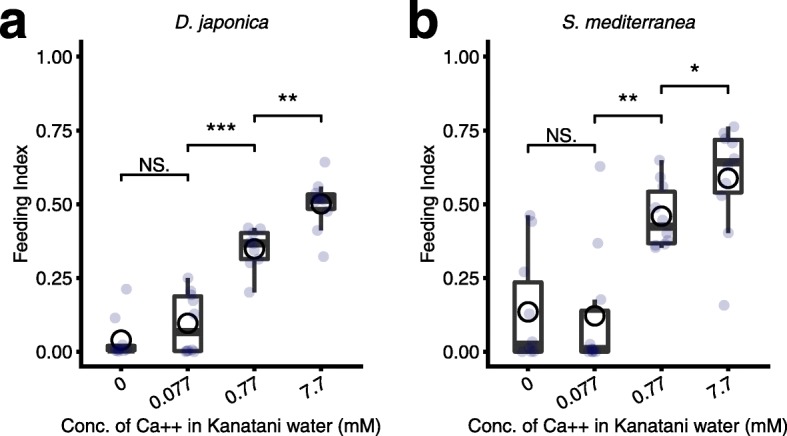
Calcium ions are required for and improve food intake. **a**. Feeding indexes of *D. japonica* in Kanatani water lacking calcium ions (Ca^++^ (−)), Kanatani water containing a low concentration of calcium ions (0.1x Ca^++^), original Kanatani water (1x Ca^++^) or Kanatani water containing excess calcium ions (10x Ca^++^). **b**. Feeding indexes of *S. mediterranea* under the same conditions as tested in *D. japonica*. *n* = 10; NS, not significant; *, *p* < 0.05; **, *p* < 0.01; ***, *p* < 0.005 (Wilcoxon test)

In order to reveal how the sensitivity of the response to foods was modulated by calcium ions in the environmental water, we performed the food-intake assay using Kanatani water containing different concentrations of calcium ions. When we used Kanatani water without calcium ions (Ca^++^ (−)) or with a low concentration of calcium ions (0.077 mM Ca^++^), the feeding index showed low scores compared to the score obtained with the original Kanatani water (0.77 mM Ca^++^) (Fig. 6a). Conversely, when we increased the concentration of calcium ions in the Kanatani water (7.7 mM Ca^++^), nearly all of the intestinal ducts of planarians were filled with the food, and planarians showed an increased score of the feeding index compared to that obtained with the original Kanatani water. Additionally, the food-intake assay using another planarian species, *S. mediterranea*, which dwells in hard waters, in contrast to *D. japonica*, which dwells in soft waters, also showed that calcium ions in the environmental water increased the food intake of *S. mediterranea* in a concentration-dependent manner (Fig. 6b), indicating that activation of feeding behavior by environmental calcium ions is independent of the planarian species or the concentration of calcium ions in the planarian’s natural habitat. These results suggest that environmental calcium ions are indispensable for and promote the feeding behavior in a concentration-dependent manner. Taken together, the present findings suggest that calcium ions in the environmental water define the responsive sensitivity of planarians to food, resulting in impacts on the feeding behavior, and consequently impacts on the population size of planarians.

## Discussion

Here we showed that the sensitivity of the responsiveness to foods in planarians is modulated by calcium ions in the environmental water. In insects, octopaminergic neurons and dopaminergic neurons in the central nervous system regulate the sensitivity to foods during feeding behavior [42–47], and in the neurons that respond to the respective amines, calcium ions, which act as a second messenger, are increased in the cytosol as a result of activation of a guanine nucleotide-binding protein (G protein) [48]. These findings suggest that deficiency of calcium in the environmental water may interfere with the activity of octopaminergic or dopaminergic neurons in the brain that regulate the feeding behavior of planarians. However, we found here that the removal of calcium ions from the environmental water immediately impaired the feeding behavior (Figs. 5, 6), suggesting that calcium ions in the environmental water are involved in the activation of the chemosensory neurons distributed in the body surface rather than brain neurons.

It has been reported that the feeding behavior of planarians occurs by sequential processes consisting of food-localization behavior and food-intake behavior by the pharynx protruded from the body [26]. Our data in the present study showed that deficiency of calcium ions in the environmental water inhibited both the food-localization and food-intake processes (Figs. 5, 6). These results suggest that similar calcium-concentration-dependent signaling machinery may be involved in these behavioral processes determining the sensitivity of the response to foods. Transient receptor potential (TRP) channels known to play critical roles in the transduction of multimodal sensory signals, including olfaction, are non-selective cationic channels, which allow for the influx of calcium ions into cells. In planarians, some TRP channel genes were identified and were found to be widely distributed in the periphery of the body and in various tissues [21, 49, 50], although no TRP channel genes expressed in chemosensory neurons in lateral branches elongated from the brain or in the pharynx in the middle of the body have been identified. These findings collectively suggest that the sensitivity of the chemosensory signaling coupled with transducers such as TRP channels may be influenced by calcium ions in the environmental water in a concentration-dependent manner, resulting in modulation of the feeding behavior in planarians.

Additionally, while planarians in 0% ASW/pure water displayed photo-orientation behavior with normal movement activity (Fig. 3b, c), planarians in modified Kanatani water lacking calcium ions showed reduced movement activity (Fig. 5d, e), suggesting that the balance of various ions, rather than calcium ions alone, may be important for producing planarians’ behaviors. By combining further data regarding behavioral traits obtained from the kinds of experiments presented here and additional data regarding molecules in the neurons regulating behaviors, we should be able to unravel the relationship between the neural system and ions in the environmental water in planarians.

Recently planarian biology has been advanced as a model system for neuroscience, as well as for regeneration and stem cell biology. However, the ecology of planarians in nature remains poorly investigated despite the importance of understanding animal physiology in particular environments, as well as its importance for determining appropriate culture conditions in the laboratory. In contrast to the dramatic effects of pollutants or toxins on planarian survival and general behaviors, the chemical properties of habitat water have been thought to have only a minor impact on planarian survival or populations [51]. In this study, we found that the population growth rate of planarians differs depending on the culture water. This observation, combined with the data of behavioral analyses, suggests that chemosensory neurons closely associated with the feeding behavior may be modulated by calcium ions in the environmental water, with resultant impacts on the growth and reproduction of planarians. These insights have implications about ethological and ecological effects that might result from differences of the concentration of calcium ions in planarians’ habitat waters, including possible restrictions of planarians’ distribution range in nature. Indeed, previous reports indicated that although planarians can survive in lakes with low levels of dissolved calcium ion, planarians tended to be abundant in lakes with high levels of this ion [52, 53].

**Fig. 7:**
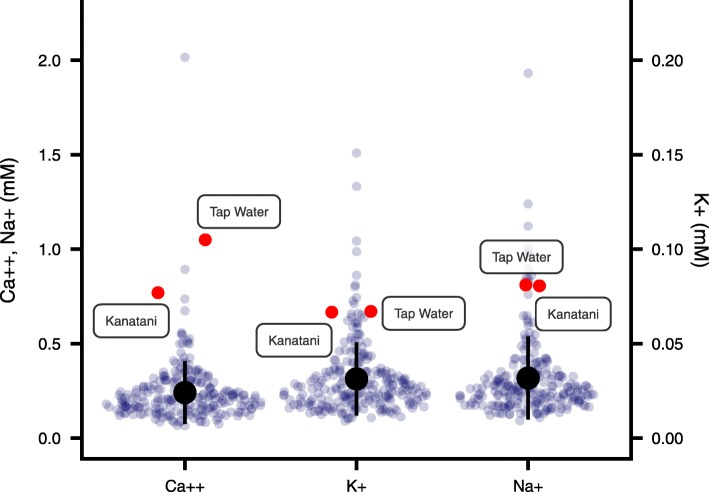
Comparison of ion concentrations among rivers in Japan and water characteristics used in this study. Concentrations of calcium, potassium, and sodium ions in rivers throughout Japan are indicated by gray dots. Concentrations of calcium, potassium, and sodium ions of tap water and Kanatani water are indicated by red dots. Both the tap water and Kanatani water contain a relatively high concentration of ions compared to those of rivers in Japan. Circles and vertical bars are mean ± sd

The ionic properties of the Kanatani water and the tap water used in this study were very similar (Fig. 7), and this is consistent with the fact that we could find no significant differences in behaviors between planarians in Kanatani water and tap water. *D. japonica* is distributed in a wide variety of rivers throughout Japan [1], whose waters are all categorized as soft water, while both the tap water used here and Kanatani water contain a relatively high concentration of ions compared to those of rivers in Japan [8, 40, 41] (Fig. 7). In other words, the ionic properties of rivers in Japan are not optimal for the planarian feeding behavior, and the feeding behavior observed in this study may have included a boosting effect resulting from a higher concentration of calcium ions.

Planarians are postulated to have originated in Gondwanaland (Africa) approximately 300 million years ago and then spread to southern Europe and eventually to have reached the Far East [54, 55]. Therefore, planarians may have initially been optimized for a habitat with a higher concentration of calcium ions, and may have adapted to lower concentrations of calcium ions as their distribution became wider, and thus the boosting of the feeding behavior by calcium ions might be a result of maintaining the ancestral phenotype. Consistent with this, the feeding behavior in *S. mediterranea*, which dwells in hard water, was also improved by excess administration of calcium ions, like that in *D. japonica* (Fig. 6).

Although the acidification of surface-waters and rain has been mitigated by environmental regulations and agreements since the 1990s, the concentration of calcium ions was increased worldwide by acid deposition in the 1960s and 70s [56, 57]. Planarians are known as a top predator in their habitats [58]; therefore, our results imply that increases of calcium ions in aqueous systems may contribute to the extinction or decline of particular animals due to excessive predation by planarians. Taken together, the present experimental findings provide insights into not only planarians’ behavioral traits but also possible ecological impacts.

## Conclusions

We demonstrated that breeding water ionic properties specifically impact feeding behavior in planarians, and the feeding behavior was suppressed when the concentration of ions in the breeding water was low, and eventually affected the population size. Quantitative analyses of the feeding behavior immediately after transferring the planarians to different environmental waters clearly showed that feeding behavior was affected by the concentrations of ions in the environmental water itself, rather than that some defect of the homeostatic state during long-term culture in breeding water affects the feeding behavior. Using defined waters, we identified calcium ions in the environmental water as ions indispensable for the feeding behavior, as well as for potentiating it via affecting the sensitivity of the responsiveness to foods. Furthermore, our results together with comparison between the chemical compositions of the laboratory breeding waters used in this study and of bodies of water in nature suggested that planarians might potentially affect biodiversity by changing the activity of their feeding behavior according to the chemical composition of the environmental water. We propose that even slight changes in environmental factors in nature may impact the ecosystem via modification of animal behavior.

## Supplementary information

**Additional file 1: Table S1.** The composition and the ion concentration of Kanatani’s water.

**Additional file 2: Figure S1.** Feeding behavior assays in tap water with or without EGTA. **a**. The food-localization assay in tap water containing 10 mM EGTA. Planarians in tap water (Ctrl) showed a preference for moving to and staying in the region with the food, whereas planarians in tap water containing 10 mM EGTA did not show such food-localization behavior. This result is consistent with the results obtained using Kanatani water. *t* = 300 s. **b**. Time spent in the target quadrant during assay of planarians in tap water containing EGTA is shown as a box-and-whisker plot with mean (circles). **c**. Speed of movement of planarians during the assay. The absence of calcium ions in the environmental water impaired the motor activity. **d**. The food-localization index is the adjusted value of the spent time in the target quadrant calculated by assuming all individuals had the same speed of movement. The adjustment was performed using the median value of the speed (0.60 mm/sec) of the planarians in tap water containing EGTA. **e**. The feeding index of planarians in tap water containing EGTA is shown as a box-and-whisker plot with means (circles). Chelation of calcium ions by EGTA in tap water reduced food intake. **, *p* < 0.01; ***, *p* < 0.005 (Wilcoxon test).

**Additional file 3: Figure S2.** Motile cilia in the peripheral epithelium are not required for the feeding behavior. **a**. Kymograph of beating cilia of planarians during a 1 s period in Kanatani water in the absence of a particular ion. Asterisks denote successive ciliary beat cycles. Absence of calcium ions, potassium ions, or sodium ions did not affect ciliary beating. **b**. Removal of the cilia from the peripheral epithelium of planarians in Kanatani water supplemented with 1% ethanol (Cilia(−)), compared to Kanatani water alone (Ctrl). Many cilia (bracket) were observed on the peripheral epithelium of control planarians, but not on that of planarians treated with 1% ethanol. Scale bar: 10 μm. **c**. The food-localization assay of planarians with or without cilia. Planarians in tap water (Ctrl) showed a preference for moving to and staying in the region with the food, whereas cilia-removed planarians did not show such food-localization behavior. *t* = 600 s. **d**. Time spent in the target quadrant during assay of planarians with or without cilia. **e**. Speed of movement of planarians during the assay. **f**. The food-localization index of planarians with or without cilia.

## Data Availability

All materials except for a primary antibody used in the present work described in the manuscript are available from standard commercial sources (Thermo Fisher Scientific, Sigma, Nacalai Tesque, or Corning) or from the corresponding author (TI).
