# Supplementary material for: Calcium ions in the aquatic environment drive planarians to food
Source: Zoological Lett. 2019 Nov 6;5:31. doi: 10.1186/s40851-019-0147-x (PMC6836377; doi:10.1186/s40851-019-0147-x)
Supplement: Supplementary file 1 — Additional file 1: Table S1. The composition and the ion concentration of Kanatani’s water. [file 40851_2019_147_MOESM1_ESM.pdf]

Table S1. The composition and the ion concentration of Kanatani's water

	(mM)	Kanatani	Ca++ (–)	K+ (–)	Na+ (–)	0.077mM Ca++	7.7mM Ca++
Composition	$CaCl_2$	0.77	0.00	0.77	0.77	0.077	7.70
	KCl	0.07	0.07	0.00	0.07	0.07	0.07
	NaCl	0.64	0.64	0.64	0.00	0.64	0.64
	$NaHCO_3$	0.17	0.17	0.17	0.17	0.17	0.17
Ion Concentration	Ca++	0.77	0.00	0.77	0.77	0.077	7.70
	K+	0.07	0.07	0.00	0.07	0.07	0.07
	Na+	0.81	0.81	0.81	0.17	0.81	0.81
	Cl-	2.25	0.71	2.18	1.61	0.86	16.11
pH		7.6					
