# Supplementary figures and images for: Calcium ions in the aquatic environment drive planarians to food
Source: Zoological Lett. 2019 Nov 6;5:31. doi: 10.1186/s40851-019-0147-x (PMC6836377; doi:10.1186/s40851-019-0147-x)

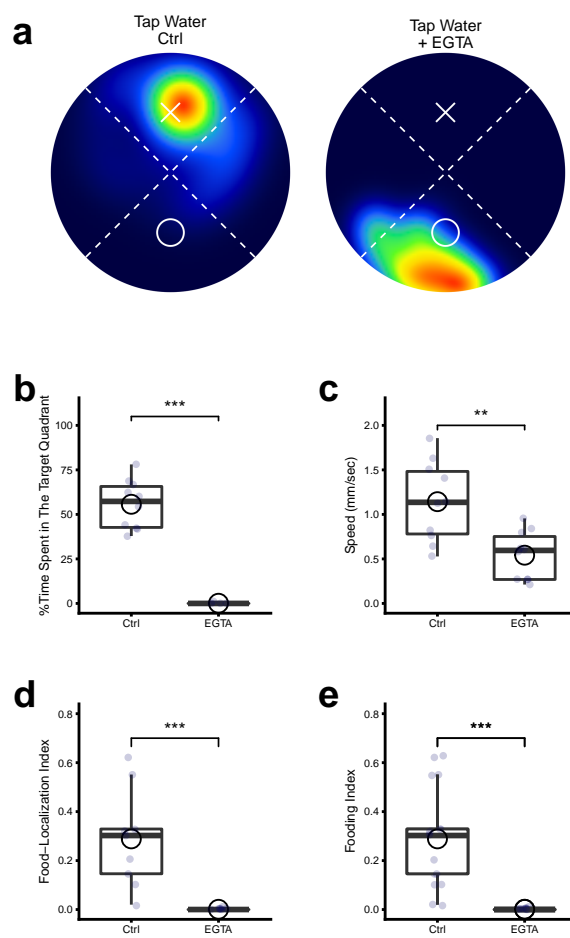

Supplement: Supplementary file 2 — Additional file 2: Figure S1. Feeding behavior assays in tap water with or without EGTA. a. The food-localization assay in tap water containing 10 mM EGTA. Planarians in tap water (Ctrl) showed a preference for moving to and staying in the region with the food, whereas planarians in tap water containing 10 mM EGTA did not show such food-localization behavior. This result is consistent with the results obtained using Kanatani water. t = 300 s. b. Time spent in the target quadrant during assay of planarians in tap water containing EGTA is shown as a box-and-whisker plot with mean (circles). c. Speed of movement of planarians during the assay. The absence of calcium ions in the environmental water impaired the motor activity. d. The food-localization index is the adjusted value of the spent time in the target quadrant calculated by assuming all individuals had the same speed of movement. The adjustment was performed using the median value of the speed (0.60 mm/sec) of the planarians in tap water containing EGTA. e. The feeding index of planarians in tap water containing EGTA is shown as a box-and-whisker plot with means (circles). Chelation of calcium ions by EGTA in tap water reduced food intake. **, p < 0.01; ***, p < 0.005 (Wilcoxon test). [file 40851_2019_147_MOESM2_ESM.pdf]

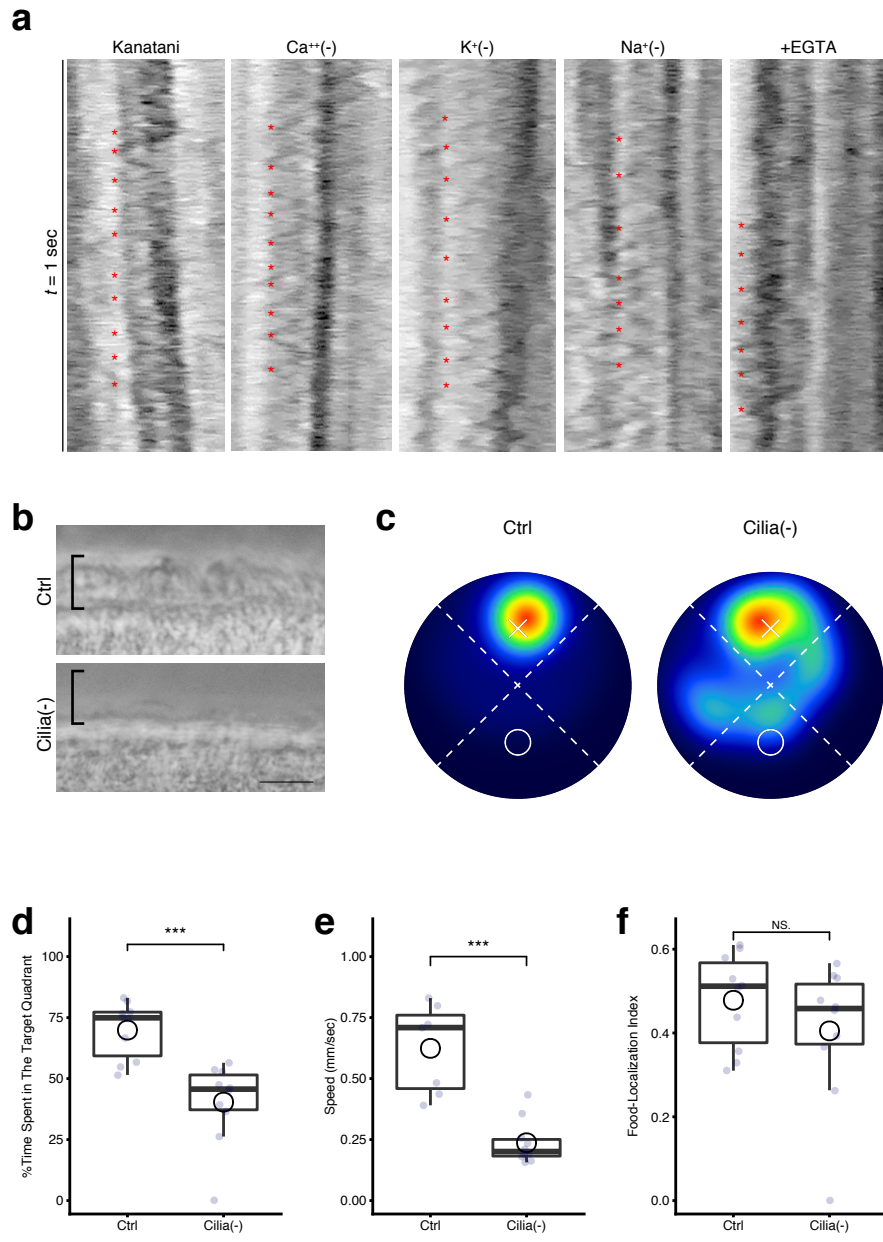

Supplement: Supplementary file 3 — Additional file 3: Figure S2. Motile cilia in the peripheral epithelium are not required for the feeding behavior. a. Kymograph of beating cilia of planarians during a 1 s period in Kanatani water in the absence of a particular ion. Asterisks denote successive ciliary beat cycles. Absence of calcium ions, potassium ions, or sodium ions did not affect ciliary beating. b. Removal of the cilia from the peripheral epithelium of planarians in Kanatani water supplemented with 1% ethanol (Cilia(−)), compared to Kanatani water alone (Ctrl). Many cilia (bracket) were observed on the peripheral epithelium of control planarians, but not on that of planarians treated with 1% ethanol. Scale bar: 10 μm. c. The food-localization assay of planarians with or without cilia. Planarians in tap water (Ctrl) showed a preference for moving to and staying in the region with the food, whereas cilia-removed planarians did not show such food-localization behavior. t = 600 s. d. Time spent in the target quadrant during assay of planarians with or without cilia. e. Speed of movement of planarians during the assay. f. The food-localization index of planarians with or without cilia. [file 40851_2019_147_MOESM3_ESM.pdf]
